# Ischemic Stroke, Major Bleeding and Death Following Oral Anticoagulation for Atrial Fibrillation Among Elderly Long-term Care Residents With Chronic Kidney Disease

**DOI:** 10.1016/j.xkme.2026.101456

**Published:** 2026-06-26

**Authors:** Ziv Harel, Yuguang Kang, Kathryn Stirling, Joel G. Ray, Sara L. Win

**Affiliations:** 1 Division of Nephrology, St. Michael’s Hospital, Toronto, Ontario, Canada; 2 ICES Ontario, Ontario, Canada; 3 Department of Medicine, St. Michael’s Hospital, Toronto, Ontario, Canada; 4 Division of Nephrology, McGill University Health Centre, Montreal, Quebec, Canada

To the Editor:

Atrial fibrillation (AF) affects 19-24% of older adults with CKD.^1^ Nearly 40% of seniors residing in a long-term care facility have CKD.^2^ Despite their heightened risk of ischemic stroke,^3^ only about 50% of long-term care residents with AF and CKD receive oral anticoagulant (OAC) therapy.^4^ One explanation is the ongoing uncertainty about the risk-benefit ratio of OAC therapy in this more frail population,^5^ who were largely excluded from randomized clinical trials. This study evaluated their risk of ischemic stroke, major bleeding, and death with OAC use.

This population-based cohort study used linked datasets (Table S1) at ICES, which is an independent nonprofit organization that analyzes data collected from Ontario’s publicly funded healthcare system. Use of data in this project was authorized under section 45 of Ontario’s Personal Information Protection Act, waiving the need for ethics review or informed consent. The study period was from January 2011 to December 2022.

Included were all adults aged ≥ 66 years old, residing in long-term care, with known CKD (defined using either an estimated glomerular filtration rate < 60 mL/min or receiving maintenance dialysis) and who had a diagnosis of AF using a validated algorithm.^6^ Excluded were non-Ontario residents, OAC use within 180 days before the AF diagnosis, or a history of kidney transplantation. (Fig S1) Newly prescribed OAC therapy, the exposure of interest, had to follow the AF diagnosis date (index date).

The primary efficacy outcome was ischemic stroke, and the primary safety outcome was major bleeding. Secondary outcomes included mortality within 30 days of a major bleeding event and all-cause mortality.

Cox proportional hazards regression generated hazard ratios (HRs) for the association between time-varying OAC use and each outcome. HRs were adjusted for known important factors for stroke and bleeding (Table S2). Follow-up was up to either an outcome event, death, OAC discontinuation, or arrival at the end of the study period of December 31, 2022. For patients receiving OAC therapy, only their first prescription was analyzed. Additionally, a Fine-Gray model determined the risk of ischemic stroke, with death as a competing event.

Included were 1010 older adults prescribed an OAC and 1770 not receiving an OAC (Table 1). Apixaban accounted for 49% of all OAC prescriptions, followed by warfarin (48%). The mean (standard deviation) age was about 87 years; 68% were female, and 70% had dementia. The mean glomerular filtration rate was about 40 mL/min, and the median CHA_2_DS_2_-VASc score was 5 (interquartile range, 4-5) (Table 1).

**Table 1:** Baseline Characteristics of the Cohort Participants

Characteristic	Entire Cohort (N=2780)	OAC (N=1010)	No OAC (N=1770)	Standardized Difference (%)
Demographics				
Age, mean (SD), y	86.7 (7.2)	86.1 (6.9)	87.1 (7.3)	14
Female sex, n (%)	1896 (68.2)	701 (69.4)	1195 (67.5)	4
Rural residence, n (%)	288 (10.4)	98 (9.7)	190 (10.7)	3
Comorbidities, within 3 y of index date, n (%)				
Congestive heart failure	881 (31.7)	343 (34.0)	538 (30.4)	8
Dementia	1880 (67.6)	639 (63.3)	1241 (70.1)	14
Diabetes	1397 (50.3)	532 (52.7)	865 (48.9)	8
Hip or pelvis fracture	217 (7.8)	75 (7.4)	142 (8.0)	2
Hypertension	2610 (93.9)	959 (95.0)	1651 (93.3)	7
Ischemic heart disease	627 (22.6)	250 (24.8)	377 (21.3)	8
Major hemorrhage	175 (6.3)	52 (5.1)	123 (6.9)	8
Myocardial infarction	193 (6.9)	72 (7.1)	121 (6.8)	1
Peripheral vascular disease	57 (2.1)	19 (1.9)	38 (2.1)	1
Ischemic stroke/TIA	349 (12.6)	140 (13.9)	209 (11.8)	6
Clinical risk scores, 1 y before the index date				
ATRIA bleeding score, median (IQR)	6 (6-6)	6 (6-6)	6 (6-7)	20
CHA_2_DS_2_VaSc score, median (IQR)	5 (4-5)	5 (4-6)	5 (4-5)	12
Frailty risk measure, mean (SD)	0.15 (0.12)	0.15 (0.12)	0.15 (0.12)	0
Laboratory markers, 1 y before the index date				
Serum creatinine level (μmol/L), mean (SD)	155 (111)	144 (95)	161 (120)	16
eGFR (mL/min min/1.73 m^2^), mean (SD)				
eGFR category (mL/min min/1.73 m^2^), n (%)	40 (14)	41 (13)	39 (14)	14
45-59	1162 (41.8)	447 (44.3)	715 (40.4)	8
30-44	903 (32.5)	339 (33.6)	564 (31.9)	4
15-29	433 (15.6)	162 (16.0)	271 (15.3)	2
<15	88 (3.2)	15 (1.5)	73 (4.1)	16
<15 (receiving dialysis)	194 (7.0)	47 (4.7)	147 (8.3)	15

*Note:* All data are shown as a number (%) unless otherwise indicated.

Abbreviations: ATRIA, anticoagulation and risk factors in atrial fibrillation; eGFR, estimated glomerular filtration rate; OAC, oral anticoagulant; TIA, transient ischemic attack.

The adjusted HR for ischemic stroke in relation to OAC use was 0.44 (95% confidence interval, 0.35-0.55), which was largely unchanged after accounting for death as a competing risk (Table 2). Conversely, although absolute rates were about half those for ischemic stroke, the adjusted HR for major bleeding was higher with OAC use (adjusted HR, 1.44; 95% confidence interval, 1.05-1.97) (Table 2).

**Table 2:** Ischemic Stroke, Major Bleeding, and All-cause Mortality in Relation to Oral Anticoagulant Use for Atrial Fibrillation Among Elderly Long-term Care Residents With CKD

Outcome	Oral Anticoagulant Use		No Oral Anticoagulant use		Study Outcomes Comparing Users vs Nonusers of an Oral Anticoagulant		
	No. With Outcome/No. at Risk (%)	Incidence Rate Per 100 Person-years	No. With Outcome/No. at Risk (%)	Incidence Rate Per 100 person-years	Unadjusted HR (95% CI)	aHR^a^ (95% CI)	Competing risk aHR^a^^,^^b^ (95% CI)
Ischemic stroke	182/1010 (18)	9.7	195/1770 (11)	14.3	0.48 (0.39-0.60)	0.44 (0.35 -0.55)	0.46 (0.42-0.51)
Major bleeding	112/1010 (11)	5.6	72/1770 (4)	5.1	1.26 (0.94-1.69)	1.44 (1.05-1.97)	—
Mortality within 30 d of major bleeding	24/1010 (2)	1.1	17/1770 (1)	1.1	1.25 (0.66-2.34)	1.41 (0.73-2.73)	—
All-cause mortality	887/1010 (88)	40.1	1705/1770 (96)	114.4	0.45 (0.41-0.49)	0.47 (0.43-0.51)	—

Abbreviations: aHR, adjusted hazard ratio; CI, confidence interval; HR, hazard ratio.

a Adjusted for age, female sex, CHA_2_DS_2_-VASc score, anticoagulation and risk factors in atrial fibrillation (ATRIA) score, congestive heart failure, dementia, diabetes mellitus, hip or pelvic fracture, frailty index, history of major hemorrhage, chronic hypertension, history of prior ischemic stroke/ transient ischemic attack (TIA), history of myocardial infarction, peripheral vascular disease, and estimated glomerular filtration rate (eGFR).

b With death handled as a competing event.

Mortality within 30 days of major bleeding was rare and not different by OAC use, whereas all-cause mortality was very common and significantly lower among OAC users (Table 2).

In this population-based cohort study of frail older adults with CKD and AF, the use of OAC was associated with a significantly lower risk of ischemic stroke and a higher risk of major bleeding. The incidence of ischemic stroke was considerably higher than that of major bleeding.

As a study limitation, unmeasured confounding could still explain the observed associations, given the many reasons why an older adult might be in a long-term care facility. Second, few patients with advanced CKD were included herein. Third, direct OAC dosing was not assessed, nor was the time in the therapeutic range among warfarin users. Fourth, antiplatelet medication use was not known. Finally, measures of functional status were not available in our dataset. However, frailty was included as a covariate, as it has been shown to influence anticoagulation decisions independent of functional status.^7^

Further study is needed to define the optimal OAC strategy in frail elderly persons with AF and advanced CKD.
