# Supplementary material for: Ischemic Stroke, Major Bleeding and Death Following Oral Anticoagulation for Atrial Fibrillation Among Elderly Long-term Care Residents With Chronic Kidney Disease
Source: Kidney Med. 2026 Jun 26;8(9):101456. doi: 10.1016/j.xkme.2026.101456 (PMC13524551; doi:10.1016/j.xkme.2026.101456)
Supplement: Supplementary file 1 [file mmc1.pdf]

SUPPLEMENTARY MATERIAL

Table S1: Description of the ICES databases used in the study.

Dataset name	Description
Canadian Institute for Health Information Discharge Abstract Database (CIHI-DAD)	Contains administrative, clinical (diagnoses and procedures/interventions), demographic, and administrative information for all admissions to acute care hospitals in Ontario. Diagnostic codes are captured using the International Statistical Classification of Diseases and Related Health Problems, 10th Revision, Canada (ICD-10-CA) coding system and interventions (up to 20 on a given DAD record) are captured using the Canadian Classification of Health Interventions (CCI) coding system.
Canadian Institute for Health Information Same Day Surgery (CIHI-SDS)	Contains administrative, clinical (diagnoses and procedures/interventions), demographic, and administrative information for all day surgeries in Ontario. Diagnostic codes are captured using the International Statistical Classification of Diseases and Related Health Problems, 10th Revision, Canada (ICD-10-CA) coding system and interventions (up to 20 on a given DAD record) are captured using the Canadian Classification of Health Interventions (CCI) coding system.
Ontario Dementia Database (DEMENTIA)	Contains all incident and prevalent cases of dementia in Ontario.
Ontario Hypertension Dataset (HYPER)	Contains all incident and prevalent cases of hypertension in Ontario.
National Ambulatory Care Reporting System (NACRS)	Contains administrative, clinical (diagnoses and procedures), demographic, and administrative information for all Ontarian patient visits made to hospital- and community-based ambulatory care centres (emergency departments, day surgery units, hemodialysis units, and cancer care clinics).
Ontario Diabetes Database (ODD)	Contains all prevalent and incident cases of diabetes in Ontario using a validated algorithm.
Ontario Drug Benefit (OBD)	Contains all outpatient prescription medications dispensed to Ontarians over the age of 65, and has an accuracy exceeding 99% in comparison to the reference standard of pharmacy chart review.[1]
Ontario Health Insurance Plan (OHIP) Claims History Database	Contains information on inpatient and outpatient services provided to Ontario residents eligible for the province's publicly funded health insurance system by fee-for-service health care practitioners (primarily physicians) and "shadow billings" for those paid through non-fee-for-service payment plans.

Ontario Laboratories Information System (OLIS)	Contains information regarding lab test orders and results from hospitals, community labs, and public health labs across Ontario.
Registered Persons Database (RPDB)	Provides basic demographic information for those issued an Ontario health insurance number, and indicates the time periods for which an individual was eligible to receive publicly funded health insurance benefits and the best-known postal code for each registrant on July 1st of each year.

Table S2. Table of variables for the exposure group, definitions used for cohort entry/exclusion criteria, study exposure, outcome, and adjustment variables.

Assessment	Timing	Disease, procedure or condition	Database	CIH ICD-10-CA or CCI codes	OHIP ICD-9 diagnostic codes or fee codes
Cohort entry criteria	Index date	Atrial fibrillation (AF) defined as: Hospitalization or an emergency room code for AF **OR** prescription for an AF specific antiarrhythmic (Propafenone, Flecainide, Sotalol, Amiodarone) **OR** billing code for cardioversion (electrical or chemical).	OHIP<br>CIHI-DAD<br>NACRS<br>ODB	I48, I480,I4800, I4801,I4802, I481,I483,I484,I4890,I4891	OHIP diagnostic: 4273, 42731, 42732<br>OHIP fee: Z437 (cardioversion)
	On or within 2 years prior to the index date	Kidney function i.e. estimated glomerular filtration rate (eGFR)	OLIS		OLIS Kidney Function defined as two outpatient eGFR measures separated by at least 90 days but no more than 365 days of each other. The most recent lab value cannot be more than a year from index date. The 2 eGFR measures must fall within 10 ml/min/1.73m2/ or <=10% of one.
Exclusion criteria	At index date	Non-Ontario resident	RPDB		
	At index date	Age >66 years	RPDB		
	At index date	Missing or invalid age/sex	RPDB		
	On or prior to the index date	Death	RPDB		
	Prior to the index date	Prior history of AF	OHIP<br>CIHI-DAD<br>NACRS<br>ODB	See cohort entry	

	365 days prior to the index date	Previous prescription for an oral anticoagulant	OLIS		
	Prior to the index date	No outpatient measurement of kidney function	OLIS		
	Prior to the index date	Kidney transplant	OHIP<br>CIHI-DAD<br>NACRS<br>ODB		S434,435
Study exposure	On or after the index date	Oral anticoagulation	OBD		
Study outcome	After the index date	Ischemic stroke /TIA	CIHI-DAD<br>NACRS	G450, G451,G452,G453,G458, G459,H340,H341,I60,I600,I601, I602,I603,I604,I605,I606,I607, I608,I609,I61,I610,I611,I612,I613, I614,I615,I616,I618,I619,I630, I631,I632,I633,I634,I635,I638, I639,I64	432, 435, 436
	After the index date	Major bleeding	CIHI-DAD<br>NACRS	ICD10: I600, I601, I602, I603, I604, I605, I606, I607, I609, I61, I62, I850, I9820, I983, K2210, K2212, K2214, K2216, K226, K228, K250, K252, K254, K256, K260, K262, K264, K266, K270, K272, K274, K276, K280, K282, K284, K286, K290, K3180, K6380, K920, K921, K5520, K625, K922<br><br>Based on a validation study demonstrating PPV and NPV of 87% and 92%.	
	After the index date	All-cause mortality	RPDB		

Covariates	At index date	Age	RPDB		
	At index date	Female sex	RPDB		
	At index date	Rural residence	RPDB		
	2 years prior to index date	Congestive Heart Failure	CIHI-DAD<br>CIHI-SDS<br>OHIP	I099,I255,I420,I425,I426,I427,I428 I429,I43,I430,I431,I432,I438,I50,I500 I501,I5010,I5011,I5019,I509,J81	1HP53,1HP53GPQP 1HP53LAQP,1HP55 1HP55GPQP,1HP55LAQP 1HZ53GRFR,1HZ53LAFR 1HZ53SYFR<br><br>OHIP: 428, R701, R702, Z429
	2 years prior to index date	Dementia	DEMENTIA database		
	2 years prior to index date	Diabetes	ODD		
	2 years prior to index date	Hip or Pelvis fracture	CIHI-DAD<br>NACRS<br>OHIP	S72000, S72001, S72010, S72011, S72080, S72081, S72090, S72091, S72100, S72101, S72110, S72111, S72120, S72121, S72190, S72191, S72200, S72201, S32100, S32101, S32300, S32301. S32400. S32401 S32500, S32501, S32700,S32701 S32800, S32801	808
	2 years prior to index date	Hypertension	HYPER database	401, 405, 642.0-642.2, 642.7 [I10, I15, O10, O11]	

2 years prior to index date	Ischemic Heart disease	CIHI-DAD<br>CIHI-SDS<br>OHIP	I20, I200, I201,I2080,I2088,I209,I21<br>I210,I211,I212,I213,I214,I2140,I2141<br>I2142,I2149,I219,I22,I220,I221,I228<br>I229,I23,I230,I231,I232,I233,I234,I23<br>5<br>I236,I2380,I2381,I2382,I2388,I2389<br>I24,I240,I241,I248,I249,I25,I250,I251<br>I2510,I2511,I2512,I2513,I2514,I2515<br>I2519,I252,I253,I254,I255,I256,I258<br>I259	OHIP: 410, 412, 413<br>G298, R742, R743, Z434
2 years prior to index date	Major Bleeding	CIHI-DAD<br>CIHI-SDS	See outcomes above	
2 years prior to index date	Myocardial Infarction	CIHI-DAD<br>CIHI-SDS	I21, I210, I211, I212, I213, I214,<br>I2140<br>I2141, I2142, I2149, I219, I22, I220,<br>I221, I228, I229	
2 years prior to index date	Peripheral Vascular Disease	CIHI-DAD<br>OHIP	I700, I702, I7020. I7021, I708, I709<br>I731, I738, I739, K551	OHIP: E626, E649, E672,<br>R780, R783, R784, R785,<br>R786, R787, R791, R794,<br>R797, R804,R809,R813,<br>R814, R815,R855, R856<br>R860,R861,R867,R875,R93<br>3<br>R934,R936, R937
2 years prior to index date	Ischemic Stroke/TIA	CIHI-DAD<br>NACRS<br>OHIP	See outcomes above	
2 years prior to index date	ATRIA bleeding score	CIHI-DAD<br>CIHI-SDS<br>OHIP		

	2 years prior to index date	CHA_2DS_2VASc Score	CIHI-DAD<br>CIHI-SDS<br>NACRS<br>OHIP<br>RPDB		
	At the index date	Frailty risk measure	CIHI-DAD	CIHI-Hospital frailty risk measure (CIHI-HFRM)[2]	
	On or 2 years prior to the index date	eGFR	OLIS		

Figure S1: Cohort assembly.

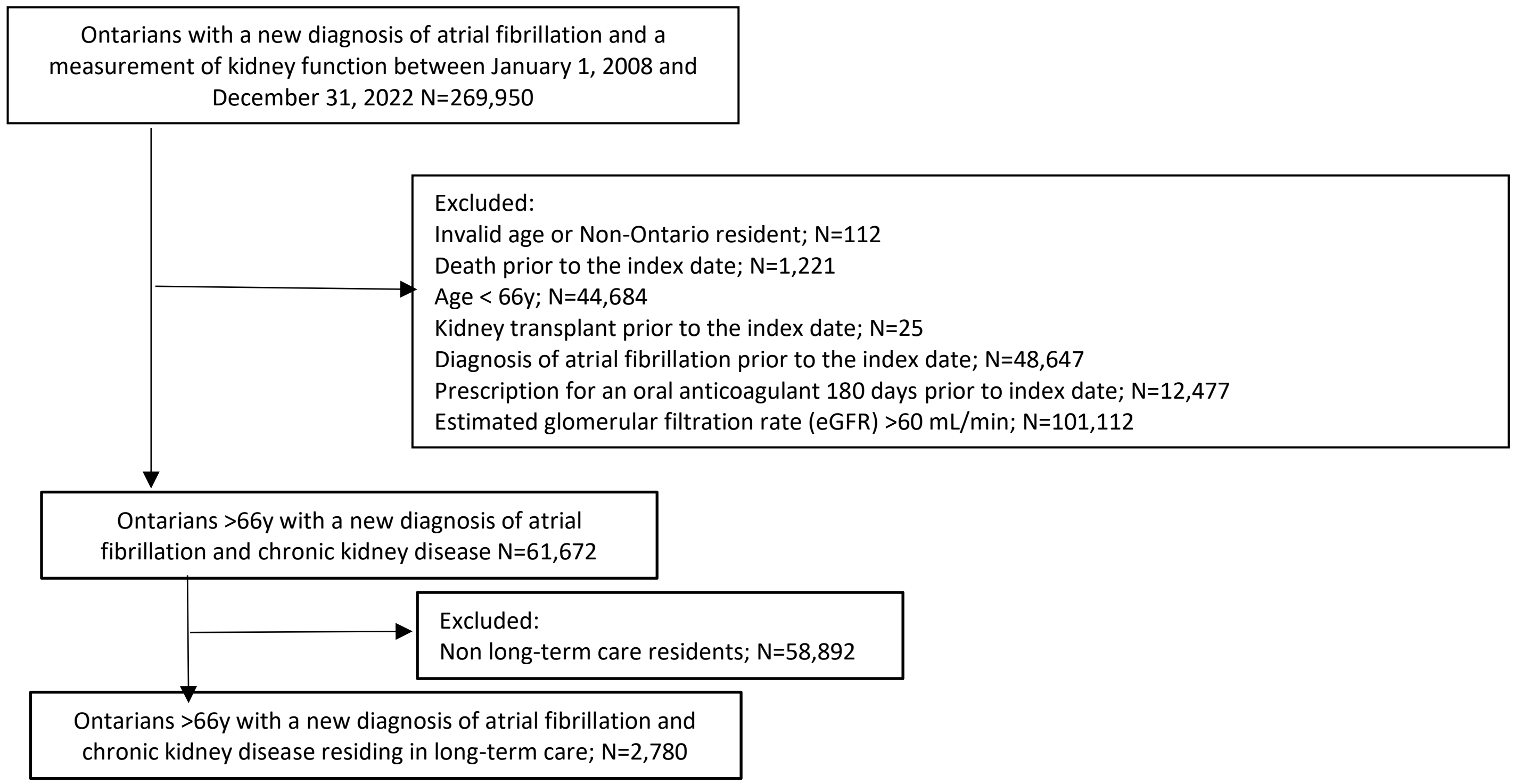

Supplementary References

1. Levy AR, O'Brien BJ, Sellors C, Grootendorst P, Willison D. Coding accuracy of administrative drug claims in the Ontario Drug Benefit database. Can J Clin Pharmacol 2003;10:67-71.
2. Amuah JE, Molodianovitsh K, Carbone S, et al. Development and validation of a hospital frailty risk measure using Canadian clinical administrative data. Cmaj 2023;195:E437-e48.
